# Hedonic and eudaimonic well-being: the role of resilience beyond fluid intelligence and personality traits

**DOI:** 10.3389/fpsyg.2015.01367

**Published:** 2015-09-11

**Authors:** Annamaria Di Fabio, Letizia Palazzeschi

**Affiliations:** Department of Education and Psychology (Psychology Section), University of FlorenceFlorence, Italy

**Keywords:** fluid intelligence, personality traits, resilience, hedonic well-being, eudaimonic well-being

## Abstract

Resilience is a key factor in the well-being of individuals. The present study set out to analyze the role of fluid intelligence, personality traits, and resilience in hedonic and eudaimonic well-being (EWB) in order to determine the incremental validity of resilience with respect to fluid intelligence and personality traits in 168 Italian high school students. The Advanced Progressive Matrices, the Big Five Questionnaire, the Connor-Davidson Resilience Scale, the Satisfaction With Life Scale, the Positive and Negative Affect Schedule, the Meaningful Life Measure, the Authenticity Scale were administered to the participants in the study. The results showed that resilience added a significant percentage of incremental variance with respect to fluid intelligence and personality traits in relation to life satisfaction, positive affect, life meaning, and authenticity. These results underline the value of resilience in both hedonic and EWB, thus offering new perspectives for research and intervention.

## Introduction

American Psychological Association (APA) guidelines (Hage et al., 2007) indicate the importance of a preventive perspective in psychological health and well-being. Preventive programs focus on reducing risk factors and increasing protective factors that can decrease the probability of negative outcomes (Hage et al., 2007; Kenny and Hage, 2009).

The preventive perspective is more effective when efforts to reduce risks are combined with efforts to increase resources (Hage et al., 2007; Kenny and Hage, 2009) aimed at building individual strengths (Steinmayr et al., 2011; Di Fabio and Kenny, 2012a,b, 2015; Di Fabio et al., 2012, in press; Di Fabio, 2014b; Di Fabio and Saklofske, 2014; Schwinger et al., 2014; Christiansen et al., 2015).

Positive psychology (Seligman and Csikszentmihalyi, 2000; Seligman, 2002; Vázquez et al., 2006; Delle Fave, 2014) involves resource promotion and the study of well-being, happiness, and mental health. In examining well-being, it distinguishes between hedonic well-being (Kahneman et al., 1999) and eudaimonic well-being (EWB; Ryan and Deci, 2001; Ryff and Singer, 2008; Waterman et al., 2010). The hedonic approach focuses on happiness, defining well-being in terms of pleasure attainment and pain avoidance (Kahneman et al., 1999). It can be seen as subjective well-being (SWB, Kahneman et al., 1999) consisting of a cognitive component of evaluation in terms of life satisfaction and an affective component characterized by the prevalence of positive emotions rather than negative emotions. The eudaimonic approach, on the other hand, relates to meaning, and self-realization where well-being is seen as the full functioning of the person (Ryan and Deci, 2001). More specifically, it can be considered psychological well-being (PWB, Ryff and Singer, 2008), also known as EWB, (Waterman et al., 2010), with the focus on resources and strengths and on life meaning, authenticity, and purposefulness (Waterman et al., 2010).

Largely because of the instability and economic turmoil that characterizes life in the 21st century, the well-being of individuals is under threat (Di Fabio et al., in press). Prevention psychologists study the phenomenon of low individual well-being and particularly the early identification of individual factors that foster well-being (Di Fabio, 2006; Hage et al., 2007; Di Fabio and Bernaud, 2008). Primary prevention focuses on positive youth development (Lerner et al., 2005), emphasizing the importance of promoting personal resources so that young people can participate productively in society (Lerner, 2001; Kenny et al., 2014). Adolescence is a critical developmental period with implications for the well-being of the individual (Call et al., 2002). Well-being in adolescence is accordingly extensively studied in the literature (Froh et al., 2009; Schlechter and Milevsky, 2010; Almquist et al., 2013). From a positive youth development perspective (PYD; Lerner et al., 2005), adolescents’ resources can be seen as resilience that promotes positive adaptation thereby enhancing well-being (Catalano et al., 2004; Lee et al., 2012).

In studies on individual characteristics in relation to well-being, intelligence has been theoretically hypothesized as playing a role (Sternberg and Grigorenko, 2004), yet no relation has been found between measures of intelligence and well-being (Sigelman, 1981; Watten et al., 1995; Wirthwein and Rost, 2011). However, studies have shown a relation between personality traits and both hedonic well-being (Gutiérrez et al., 2005; James et al., 2012) and EWB (Lavigne et al., 2013; Işık and Üzbe, 2015).

Research has also shown that resilience is a key individual characteristic in the well-being of individuals (Samani et al., 2007; Abolghasemi and Taklavi Varaniyab, 2010; Souri and Hasanirad, 2011; Liu et al., 2012; He et al., 2013; Smith and Hollinger-Smith, 2015). Resilience refers to the ability of individuals to face and overcome adversity adaptively (Luthar et al., 2000; Campbell-Sills and Stein, 2007) and, more specifically, to the implementation by individuals of adaptation strategies to cope with discomfort and adversity (Tugade and Fredrickson, 2004). Resilience also refers to an array of resources that enable individuals to transform critical experiences into opportunities for personal growth (Malaguti, 2005). Resilience is a particularly promising individual resource as it can be increased through specific training (Peng et al., 2014; Zamirinejad et al., 2014; Di Fabio et al., in press).

The literature shows a positive relation between resilience and hedonic well-being (Samani et al., 2007; Abolghasemi and Taklavi Varaniyab, 2010; Liu et al., 2012) as well as between resilience and EWB (Souri and Hasanirad, 2011; He et al., 2013; Smith and Hollinger-Smith, 2015). Resilience can therefore be considered a promising variable in both hedonic and EWB.

### Aim and Hypotheses

The theoretical framework discussed above shows that resilience is an important preventive and protective factor in fostering positive youth development (Hage et al., 2007; Kenny and Di Fabio, 2009; Kenny et al., 2014; Di Fabio and Kenny, 2015; Di Fabio et al., in press). Resilience is also a key individual resource associated with hedonic as well as EWB (Samani et al., 2007; Abolghasemi and Taklavi Varaniyab, 2010; Souri and Hasanirad, 2011; Liu et al., 2012; He et al., 2013; Smith and Hollinger-Smith, 2015). This notwithstanding, there are no references in the literature to studies that have simultaneously analyzed resilience in relation to hedonic well-being and EWB. The present study was also the first to examine, at the same time, the contribution of resilience to both hedonic and EWB by controlling for fluid intelligence as well as personality traits. More specifically, it investigated the contribution of resilience to both hedonic and EWB, beyond fluid intelligence and personality traits, in Italian high school students in their final year of school. The particular high school students were chosen as research subjects because they were at a critical stage of their lives at the end of high school where they had to make significant choices regarding their lives and careers. Faced with impending transition and their first critical career decision, coupled with social pressure to make a choice, they could experience pressure that could decrease their individual well-being in terms of both hedonic well-being and EWB.

This study is important in relation to both hedonic and EWB because it is the first to examine whether resilience can contribute to such well-being beyond other personality traits that are considered stable in the literature (Costa and McCrae, 1985). In addition, resilience can be increased through specific training (Peng et al., 2014; Zamirinejad et al., 2014).

Against this background, we formulated the following two hypotheses.

H1: Resilience will add significant incremental variance beyond that accounted for by fluid intelligence and personality traits in relation to hedonic well-being [life satisfaction and positive affect (PA)].
H2: Resilience will add significant incremental variance beyond that accounted for by fluid intelligence and personality traits in relation to EWB (life meaning and authenticity).

## Materials and Methods

### Participants

One hundred and sixty-eight high school students in their final year in a Tuscany school system, representing 92% of all such students, participated in the study. All high school students in their final year of the school system were invited to take part. Regarding gender, 62 (36.90%) of the participants were males and 106 (63.10%) were females. The participants in the study attended college preparatory high schools of four types: scientific, classical, linguistic, and socio-pedagogical. In Italy, particularly in respect of the last three types of college preparatory high schools, there is a higher percentage of females. Thus the higher percentage of females in the study reflects merely the higher percentage of females attending these types of college preparatory high schools in Italy. The participants’ ages ranged from 18 to 20 years (*M* = 19.53, *SD* = 0.59).

### Measures

#### Advanced Progressive Matrices (APM)

The Italian version by Di Fabio and Clarotti (2007) of the Advanced Progressive Matrices (APM) test by Raven (1962) was used to evaluate fluid intelligence. The test consists of two series of items: Series I with12 items, and Series II with 36 items. The participants were asked to indicate only one response for each item, choosing from eight alternatives. The Cronbach’s alpha coefficient for the present study was 0.91.

#### Big Five Questionnaire (BFQ)

The Big Five Questionnaire (BFQ, Caprara et al., 1993) was used to evaluate personality traits. The questionnaire consists of 132 items with response options on a 5-point Likert scale ranging from 1 = *Absolutely false* to 5 = *Absolutely true*. The questionnaire enables the detection of five personality traits. The Cronbach’s alpha coefficients were 0.81 for Extraversion, 0.73 for Agreeableness, 0.81 for Conscientiousness, 0.90 for Emotional Stability, and 0.75 for Openness.

#### Connor-Davidson Resilience Scale (CD-RISC)

The Italian version by Di Fabio and Palazzeschi (2012) of the Connor-Davidson Resilience Scale (CD-RISC, Campbell-Sills and Stein, 2007) was used to evaluate resilience. The CD-RISC consists of 10 items with response options on a 5-point Likert scale ranging from *Not true at all* (0) to *True nearly all the time* (4). The confirmatory factor analysis showed a one-dimensional structure of the Italian version of the CD-RISC. The Cronbach’s alpha coefficient was 0.89.

#### Satisfaction with Life Scale (SWLS)

The Italian version by Di Fabio and Busoni (2009) of the Satisfaction with Life Scale (SWLS, Diener et al., 1985) was used to evaluate life satisfaction. The questionnaire has five items on a 7-point Likert scale ranging from 1 = *Strongly disagree* to 7 = *Strongly agree*. The confirmatory factor analysis showed a one-dimensional structure of the Italian version of the SWLS. The Cronbach’s alpha coefficient was 0.88.

#### Positive and Negative Affect Schedule (PANAS)

The Italian version by Terracciano et al. (2003) of the Positive Affect Scale of the Positive and Negative Affect Schedule (PANAS, Watson et al., 1988) was used to evaluate PA. The PANAS consists of a list of 20 adjectives of which 10 refer to PA (e.g., enthusiastic, interested, determined), and 10 refer to negative affect (NA; e.g., afraid, upset, distressed). Subjects are asked to indicate the intensity with which they generally feel on a Likert scale ranging from 1 = *Very slightly or not* at all to 5 = *Extremely*. The Cronbach’s alpha coefficient was 0.72.

#### Meaningful Life Measure (MLM)

The Italian version by Di Fabio (2014c) of the Meaningful Life Measure (MLM, Morgan and Farsides, 2009) was used to evaluate life meaning. The questionnaire consists of 23 items with a response format on a 7-point Likert scale ranging from 1 = *Strongly disagree* to 7 = *Strongly agree*. The scale detects the five dimensions of life meaningfulness: Exciting life, Accomplished life, Principled life, Purposeful life, and Valued life. The Cronbach’s alpha coefficient for the total score was 0.81. In the present study, we used the total score of the MLM, supported by its good internal consistency, to compare the results more meaningfully.

#### Authenticity Scale (AS)

The Italian version by Di Fabio (2014a) of the Authenticity Scale (AS; Wood et al., 2008) was used to evaluate authenticity. The scale consists of 12 items with a response format on a 7-point Likert scale ranging from 1 = *Does not describe me at all* to 7 = *Describes me very well*. The scale has three dimensions: Self-alienation, Authentic living, and Accepting external influence. The Cronbach’s alpha coefficient for the total score was 0.80. In the present study, we used the total score of the AS, supported by its good internal consistency, to compare the results more meaningfully.

### Procedure and Data Analysis

The tools were administered in groups in the classrooms by trained psychologists. The administration sequence was alternated to counter the possible effects of the presentation of the tools during their administration. The tools were administered at a time set by the school and in accordance with the requirements of privacy and informed consent as stipulated in Italian law (Law Decree DL-196/2003). Regarding ethical standards for research, the study followed the latest version of the Declaration of Helsinki revised in Fortaleza (World Medical Association [WMA], 2013).

Descriptive statistics were used and Pearson’s *r* correlation as well as hierarchical regressions were carried out. When the data were checked for missing values, it was found that the rate of the missing values was 3%. These values were replaced using the regression method. Gender differences were investigated in respect of the studied variables by doing separate regressions for gender, but no differences were found. Accordingly, only regressions for the entire sample are reported in the study.

## Results

Means, *SD*, and correlations between APM, BFQ, CD-RISC, SWLS, PANAS PA, MLM, and AS are reported in **Table 1**.

**Table 1:** Means, *SD*, and correlations relative to Fluid intelligence, Personality traits, Resilience, Life satisfaction, Positive affect (PA), Life meaning, and Authenticity.

	*M*	*SD*	1	2	3	4	5	6	7	8	9	10	11
(1) Fluid intelligence	36.04	7.41	-										
(2) Extraversion	74.78	9.66	0.04	-									
(3) Agreeableness	77.40	10.28	0.05	0.08	-								
(4) Conscientiousness	77.65	9.05	0.06	0.23^∗∗^	0.06	-							
(5) Emotional Stability	62.98	14.73	0.07	0.06	0.28^∗∗^	0.07	-						
(6) Openness	78.42	8.19	0.08	0.38^∗∗^	0.32^∗∗^	0.34^∗∗^	0.11	-					
(7) Resilience	24.24	6.08	0.09	0.37^∗∗^	0.15^∗^	0.27^∗∗^	0.35^∗∗^	0.34^∗∗^	-				
(8) Life satisfaction	20.61	6.10	0.10	0.35^∗∗^	0.09	0.16^∗^	0.16^∗^	0.20^∗∗^	0.62^∗∗^	-			
(9) PA	33.38	5.69	0.08	0.39^∗∗^	0.11	0.17^∗^	0.18^∗^	0.18^∗^	0.66^∗∗^	0.38^∗∗^	-		
(10) Life meaning	101.03	16.89	0.08	0.33^∗∗^	0.08	0.15^∗^	0.15^∗^	0.13^∗^	0.57^∗∗^	0.42^∗∗^	0.45^∗∗^	-	
(11) Authenticity	48.52	7.81	0.07	0.31^∗∗^	0.06	0.13^∗^	0.13^∗^	0.11^∗^	0.55^∗∗^	0.39^∗∗^	0.41^∗∗^	0.49^∗∗^	-

*N = 168. ^∗^*p* < 0.05, ^∗∗^*p* < 0.01.*

The results of four different hierarchical regression models are presented alternately with life satisfaction, PA, life meaning, and authenticity as the criterion measures and with fluid intelligence at the first step, personality traits at the second step, and resilience at the third step (**Table 2**).

**Table 2:** Hierarchical regression.

	Life satisfaction	PA	Life meaning	Authenticity
	β	β	β	β
**Step 1**				
Fluid intelligence	0.11	0.09	0.09	0.08
**Step 2**				
Extraversion	0.40^∗∗∗^	0.43^∗∗∗^	0.39^∗∗∗^	0.37^∗∗∗^
Agreeableness	0.11	0.13	0.09	0.07
Conscientiousness	0.17^∗^	0.18^∗^	0.16^∗^	0.14^∗^
Emotional stability	0.17^∗^	0.19^∗^	0.16^∗^	0.14^∗^
Openness	0.21^∗∗^	0.19^∗^	0.15^∗^	0.12^∗^
**Step 3**				
Resilience	0.51^∗∗∗^	0.55^∗∗∗^	0.48^∗∗∗^	0.44^∗∗∗^
*R^2^ Step 1*	0.02	0.01	0.01	0.01
*ΔR^2^ Step 2*	0.30^∗∗∗^	0.33^∗∗∗^	0.31^∗∗∗^	0.30^∗∗∗^
*ΔR^2^ Step 3*	0.16^∗∗∗^	0.19^∗∗∗^	0.14^∗∗∗^	0.12^∗∗∗^
*R^2^ total*	0.48^∗∗∗^	0.53^∗∗∗^	0.46^∗∗∗^	0.43^∗∗∗^

*The contributions of Fluid intelligence (First step), Personality traits (Second step), and Resilience (Third step) to Life satisfaction, PA, Life meaning, and Authenticity.*

*N = 168. ^∗^*p* < 0.05, ^∗∗^*p* < 0.01, ^∗∗∗^*p* < 0.001.*

For life satisfaction, at the first step, fluid intelligence did not account for any variance (*R*^2^ = 0.02, n.s.). At the second step, personality traits accounted for 30% of the variance, and, at the third step, resilience added 16% of incremental variance. The model overall accounted for 48% of the variance.

For PA, at the first step, fluid intelligence did not account for any variance (*R*^2^ = 0.01, n.s.). At the second step, personality traits accounted for 33% of the variance, and, at the third step, resilience added 19% of incremental variance. The model overall accounted for 53% of the variance.

For life meaning, at the first step, fluid intelligence did not account for any variance (*R*^2^ = 0.01, n.s.). At the second step, personality traits accounted for 31% of the variance, and, at the third step, resilience added 14% of incremental variance. The model overall accounted for 46% of the variance.

For authenticity, at the first step, fluid intelligence did not account for any variance (*R*^2^ = 0.01, n.s.). At the second step, personality traits accounted for 30% of the variance, and, at the third step, resilience added 12% of incremental variance. The model overall accounted for 43% of the variance.

To check for possible effects of multicollinearity, we also applied relative weights analyses to examine the variance explanation of resilience irrespective of its correlations with the personality traits. The variance explained by resilience was 0.15 (*p* < 0.001) in relation to life satisfaction, 0.18 (*p* < 0.001) in relation to PA, 0.13 (*p* < 0.001) in relation to life meaning, and 0.11 (*p* < 0.001) in relation to authenticity.

## Discussion

Considering resilience as a preventive resource (Hage et al., 2007; Kenny and Hage, 2009; Di Fabio et al., in press), the aim of the present study was to analyze the role of resilience, after controlling for the effects of fluid intelligence and personality traits, in both hedonic and EWB.

The first hypothesis was confirmed as resilience added significant incremental variance beyond that accounted for by personality traits in relation to hedonic well-being in terms of life satisfaction and PA.

The results of the present study confirmed the relation – found in the literature (Samani et al., 2007; Abolghasemi and Taklavi Varaniyab, 2010; Liu et al., 2012) – between resilience and hedonic well-being showing that individuals who perceived themselves to be more able to face and overcome adversity adaptively (Luthar et al., 2000; Campbell-Sills and Stein, 2007) experienced greater global satisfaction with their lives and more positive emotions.

The second hypothesis was also confirmed as resilience added significant incremental variance beyond that accounted for by personality traits in relation to EWB in terms of life meaning and authenticity. These results are in line with the positive association of resilience with EWB reported in the literature (Souri and Hasanirad, 2011; He et al., 2013; Smith and Hollinger-Smith, 2015) showing that resilience in this study was related to optimal functioning, particularly with regard to life meaning and authentic sense of one’s own life.

The present study was the first research endeavor to examine simultaneously the contribution of resilience to hedonic well-being as well as EWB by controlling for fluid intelligence and personality traits. Regarding intelligence, the results of the study revealed no relation between fluid intelligence and well-being, as emerged in the literature (Sigelman, 1981; Watten et al., 1995; Wirthwein and Rost, 2011). Regarding personality, the results of the study confirmed the relation between personality traits and both hedonic well-being (Gutiérrez et al., 2005; James et al., 2012) and EWB (Lavigne et al., 2013; Işık and Üzbe, 2015) as also indicated in the literature. In particular, the study highlighted a stronger association between resilience and hedonic well-being than EWB as resilience is considered the ability of individuals to face and overcome adversity adaptively (Campbell-Sills and Stein, 2007) and to increase life satisfaction and PA. The study also revealed an association between resilience and EWB, indicating the role of resilience in relation to well-being in respect also of life meaning (Morgan and Farsides, 2009) and authenticity (Wood et al., 2008).

The results of the study do indeed show the role of resilience in both hedonic and EWB, yet the limitations of the study should be noted. The research was restricted to a group of Italian high school students in a Tuscan school system who were not representative of Italian high school students in general. Future studies should therefore include participants more representative of all Italian high school students, as well as other geographical areas. It would also be useful for future research to use samples from other international contexts. A further limitation of the present study was the use of only the fluid intelligence measure. With regard to the evaluation of intelligence, future studies should consider other intelligence scales as well. A future research option could be the study of resilience in relation to other EWB variables such as the subjective experience of eudaimonia (Waterman et al., 2010), existential fulfillment (Längle et al., 2003), and flourishing (Diener et al., 2010).

If future research confirms the results of the present study, opportunities for intervention could be opened up. As resilience is a characteristic that can be increased through specific training (Peng et al., 2014; Zamirinejad et al., 2014), interventions could be planned to strengthen individual resilience in terms of a preventive perspective (Hage et al., 2007; Kenny and Hage, 2009; Steinmayr et al., 2011; Di Fabio and Saklofske, 2014; Christiansen et al., 2015; Di Fabio et al., in press) and a positive psychology perspective (Seligman and Csikszentmihalyi, 2000; Seligman, 2002) in order to promote individuals’ subjective and PWB. This is particularly important in adolescence as it is a critical period in an individual’s development (Call et al., 2002; Almquist et al., 2013). Focusing on well-being in adolescence can also promote health and well-being in adulthood (Hoyt et al., 2012).

## Conflict of Interest Statement

The authors declare that the research was conducted in the absence of any commercial or financial relationships that could be construed as a potential conflict of interest.
